# Epigenetic insights into the domestication of tetraploid peanut

**DOI:** 10.1093/plphys/kiaf254

**Published:** 2025-06-13

**Authors:** Hang Yu, Chong Zhang, Chao Zhang, Yan Shi, Yajie Xue, Liang Xie, Haifeng Wang

**Affiliations:** Key Laboratory of Horticultural Crops Biology and Germplasm Enhancement in Southwest, Ministry of Agriculture and Rural Affairs, Horticulture Research Institute, Sichuan Academy of Agricultural Sciences, Chengdu, Sichuan 610066, China; State Key Laboratory for Conservation and Utilization of Subtropical Agro-bioresources, College of Agriculture, Guangxi University, Nanning 530004, China; Key Laboratory of Ministry of Education for Genetics, Breeding and Multiple Utilization of Crops, Key Laboratory of Ministry of Agriculture and Rural Areas for Biological Breeding/College of Agriculture, Fujian Agriculture and Forestry University, Fuzhou 350002, Fujian, China; State Key Laboratory for Conservation and Utilization of Subtropical Agro-bioresources, College of Agriculture, Guangxi University, Nanning 530004, China; State Key Laboratory for Conservation and Utilization of Subtropical Agro-bioresources, College of Agriculture, Guangxi University, Nanning 530004, China; State Key Laboratory for Conservation and Utilization of Subtropical Agro-bioresources, College of Agriculture, Guangxi University, Nanning 530004, China; State Key Laboratory for Conservation and Utilization of Subtropical Agro-bioresources, College of Agriculture, Guangxi University, Nanning 530004, China; State Key Laboratory for Conservation and Utilization of Subtropical Agro-bioresources, College of Agriculture, Guangxi University, Nanning 530004, China; Yazhouwan National Laboratory, Sanya, Hainan 572025, China

## Abstract

Polyploidization is a crucial evolutionary mechanism driving species domestication that promotes species formation and adaptation by providing additional genetic information to accelerate the functional differentiation of genes and evolution of new traits. Despite its importance as a key epigenetic modification, the role of DNA methylation in polyploid domestication through the regulation of gene expression remains unclear. Here, we performed whole-genome bisulfite sequencing and RNA-seq analysis on the cultivated allotetraploid peanut (*Arachis hypogaea* L.) and its 2 ancestral diploids to investigate the epigenetic regulatory mechanisms of DNA methylation in the formation of peanut polyploids and in peanut domestication. Our findings unveiled substantial differences in DNA methylation between peanut subgenomes, particularly in non-CG contexts. Specifically, CHG methylation is a key factor regulating the expression bias of homoeologs and dominant subgenome expression, as well as stress-responsive gene expression. Additionally, CHH methylation plays a role in peanut seed development by regulating genes associated with fatty acid biosynthesis and lipid metabolism. In conclusion, our study provides a vital theoretical foundation and perspective on the epigenetics underlying cultivated peanut domestication, especially related to the formation of agronomic traits.

## Teaser

DNA methylation influences homoeologous gene expression bias during tetraploid peanut domestication.

## Introduction

Whole-genome duplication (WGD) or polyploidization is an important driving force in species evolution and exists in eukaryotes, including animals, plants, and fungi. Extensive studies have suggested that WGDs are widespread in plants and have played an essential role in species diversification and environmental adaptation (Soltis et al. 2014; Wendel et al. 2016; Wang et al. 2023). WGD could duplicate all genes on the whole set of chromosomes, resulting in a large number of duplicated genes. These redundant genes provide more genetic material for the evolution of species, buffer the impact of harmful mutations, and facilitate the functional differentiation of duplicated genes. For example, tetraploid *Citrus limonia* has better drought resistance than its diploid parents (Allario et al. 2013). Allopolyploid of white clover (*Trifolium repens*) promotes its expansion into additional ecosystems (Griffiths et al. 2019). However, WGD can also suffer some adverse effects, including changes in cell volume, spatial composition, alterations in cell structure, and physiological disturbances, and may also produce aneuploid cells. It also affects gene expression networks and produces some genetic and epigenetic changes. There are 2 types of polyploids, one of which is autopolyploid from the genome doubling of the same species, and the other is allopolyploid from the genome duplication of 2 or more diploid genomes from different species. Allopolyploids are common, such as wheat, cotton (*Gossypium hirsutum*), and oilseed rape (*Brassica napus*). In the process of polyploidy, expression bias and dominant expression of homoeologous genes may occur due to the difference in the expression of some homoeologous gene pairs (Grover et al. 2012). These phenomena may be caused by the interactions between subgenomes and the effects of epigenetic changes (Comai 2005; Song et al. 2017), which may lead to changes in crop phenotypes and traits.

DNA methylation, as one of the most conserved epigenetic modifications, plays an important role in gene expression regulation and maintenance of transposable element (TE) silencing (Wang et al. 2015, 2019; Sun et al. 2024), thus participating in many important biological processes (Gehring and Henikoff 2007; Ausin et al. 2016). In animals, DNA methylation usually occurs only on CG sequences, while in plants, DNA methylation exists in 3 different forms, namely, CG, CHG (H = A, T, or C), and CHH (Niederhuth and Schmitz 2014). In *Arabidopsis thaliana*, METHYLTRANSFERASE1(MET1), homologous to mammalian DNA methyltransferase 1 (Dnmt1), mainly maintains CG methylation (Finnegan et al. 1998) and CHROMOMETHYLASE3(CMT3) mainly maintains CHG methylation (Lindroth et al. 2001; Du et al. 2012). DOMAINS REARRANGED METHYLTRANSFERASE2 (DRM2) and CHROMOMETHYLASE2 (CMT2) (Zemach et al. 2013) maintain CHH methylation around chromosome arms and centromeres, respectively (Zemach et al. 2010; Stroud et al. 2014). In addition, DNA methylation is established by DRM2 (Cao and Jacobsen 2002a, b) through a complex pathway known as RNA-directed DNA methylation (RdDM) (Law and Jacobsen 2010; Matzke and Mosher 2014). Prior studies have shown that epigenetic modification plays an important role in the expression of homoeologous genes during plant polyploidy. For example, epigenetic modifications, such as DNA methylation and histone, play an important role in the separation and merging of the polyploid wheat genome, and changes in DNA methylation are accompanied by expression differentiation of TEs and homoeologous genes, thus affecting species selection, adaptation, and other phenotype variation. A similar phenomenon was also observed in the polyploidization of cultivated cotton (Song et al. 2017).

Peanut (*Arachis hypogaea* L.) is one of the important oil crops and cash crops. The oil content of mature peanut seeds can be as high as 50% (Kumar et al. 2019), much higher than that of soybean, which is also an important oil crop (the oil content of soybean seeds is about 20%). Moreover, peanut serves as a model species for exploring polyploid evolution and crop domestication. The allotetraploid peanut, *A. hypogaea* (Ah, 2*n* = 4*x* = 20) (Bertioli et al. 2019), emerged around 9,400 yr ago through interspecific hybridization involving 2 diploid species, *Arachis duranensis* (Ad, 2*n* = 2*x* = 10) and *Arachis ipaensis* (Ai, 2*n* = 2*x* = 10) (Bertioli et al. 2016), where the A and B subgenomes of Ah evolved from Ad and Ai, respectively. While numerous studies have focused on the expression of homoeologous genes in polyploid peanuts, the epigenetic modification mechanism underlying the variation in homoeologous gene expression and the dominance of expression compared to ancestral diploids remains elusive. Here, we performed a single-base resolution DNA methylation comparison between tetraploid peanuts and their 2 ancestor diploid peanuts using whole-genome bisulfite sequencing (WGBS). By integrating this data with transcriptome sequencing information, we were able to analyze the association between DNA methylation variance and the bias in homoeologous gene expression, as well as the dominance in expression level. The findings from this research will offer a unique and valuable epigenetic insight into the evolution of subgenomes and homoeologs in allopolyploid peanuts.

## Results

### DNA methylation landscape between diploid and tetraploid peanuts

Substantial progress has been made in peanut genomics research, highlighted by the development of a chromosome-scale reference genome for tetraploid peanuts and ancestral diploid peanuts (Bertioli et al. 2016; Yin et al. 2018; Bertioli et al. 2019). To investigate DNA methylation variations between tetraploid and diploid peanuts, we conducted a comprehensive analysis of single-base resolution DNA methylation landscape in leaf tissues (mature leaves of seedling peanuts) of 3 peanut species through WGBS, with each sample having 2 biological replicates. We obtained 1.2 billion 150-bp reads, which were aligned to the respective reference genomes. Over 0.78 billion reads were uniquely mapped to the genome, resulting in a 99.3% conversion rate (Supplementary Table S1). Additionally, the 2 biological replicates showed highly correlated coefficients of DNA methylation across different sequence contexts (Supplementary Fig. S1). This result demonstrates the high reproducibility and accuracy of our sequencing data. We also aligned reads from diploid peanuts to the 2 subgenomes of tetraploid peanuts and found that significant reduction of mapping ratio indicating that the genome sequence of tetraploid peanuts had striking variation compared to the diploid peanut genome (Supplementary Table S2). These data provide insight for the study of DNA methylation in phylogenetic closed species.

**Figure 1.**
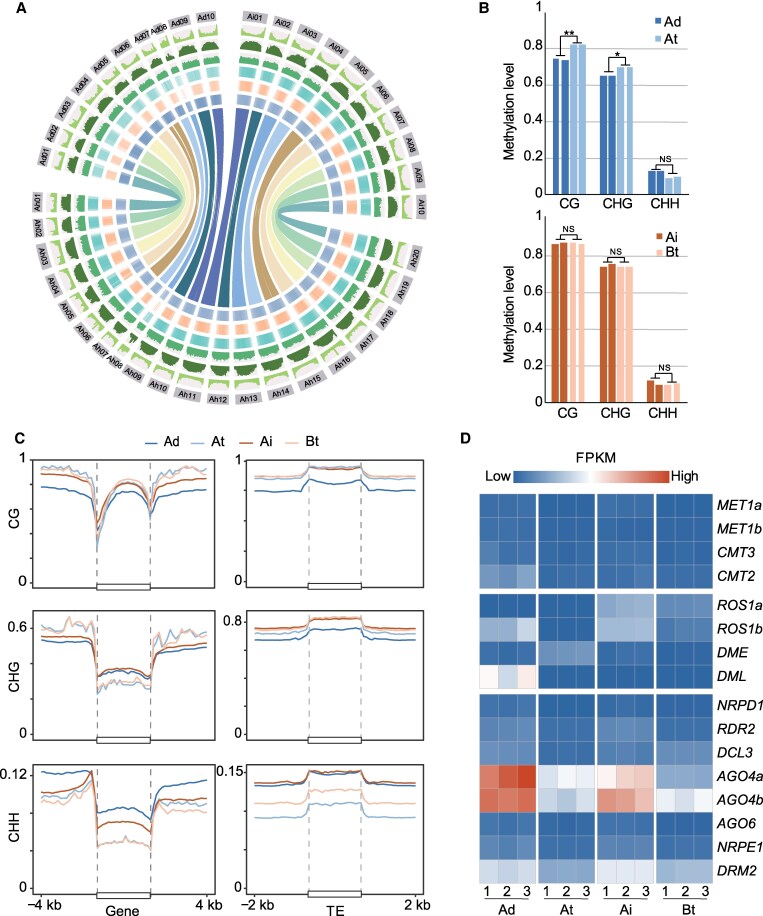
DNA methylome landscape of diploid peanuts (*A. duranensis and A. ipaensis*) and cultivated tetraploid peanut (*A. hypogaea*). **A)** Circle plots showing DNA methylome profiles of 3 peanut species, from outside to inside: chromosomes; gene density; density of LTRs; density of DNA type of TEs; CG, CHG, and CHH methylation levels; and collinear genes. **B)** Histogram showing genome-wide methylation levels per biological replicate. * and ** indicate *P* < 0.05 and 0.01 for Student's *t*-test, respectively. **C)** Metaplot showing DNA methylation patterns of genes and TEs in 3 peanut species. **D)** Heatmap showing the expression level of genes in the DNA methyltransferase, demethylase, and RdDM pathways. Ai and Ad represent the genomes of 2 diploid peanuts. At and Bt represent the A and B subgenomes of *A. hypogaea*, respectively. TE, transposable element; FPKM, fragments per kilobase of exon model per million mapped fragments.

Similar to other plant DNA methylation profiles, peanut DNA methylation is notably concentrated in peri-centromere regions, displaying consistency across all 3 peanut species (Fig. 1A). Within the A subgenome, CG and CHG methylation levels were significantly higher (*t*-test *P* < 0.05) in tetraploid peanut compared to diploid peanut, while CHH methylation was lower in tetraploid. Conversely, no significant differences were observed in CG and CHG methylation in the B subgenome, and the changes in CHH methylation mirrored those in the A subgenome (Fig. 1B). Subsequently, we examined the DNA methylation patterns in gene and TE regions. The comparison of DNA methylation in gene and flanking regions revealed patterns consistent with other plant studies, illustrating enriched CG methylation in gene body regions, higher DNA methylation in up- and downstream regions, and a significant decrease in the region of transcription start site (TSS) and transcription termination site (TTS) with all 3 sequence contexts. For TE regions of the genome, tetraploid peanuts showed increased DNA methylation compared with diploid peanuts in the CG and CHG sequence contexts, while displaying reduced CHH methylation compared with diploids. In gene and flanking regions, tetraploid peanuts showed increased CG and decreased CHH methylation compared with diploids, consistent with the global analysis. Unexpectedly, CHG methylation showed a complex trend, with diploid higher levels of CHG methylation than tetraploid in the gene regions and the opposite in the flanking regions (Fig. 1C). This result suggested that TE methylation mainly shaped the DNA methylation landscape among different peanuts, even though gene methylation diverges.

To investigate the regulatory mechanism governing changes in DNA methylation during peanut evolution by methyltransferases and demethylases, we conducted sequence comparisons with identified homologous genes in *A. thaliana*. Combining with genomic annotation, we identified DNA methyltransferases, demethylases, and RdDM pathway-related genes in 3 peanuts. Key methyltransferase responsible for maintaining CG (*MET1*), CHG (*CMT3*), and CHH (*CMT2*) methylation levels did not exhibit significant expression differences between diploid and tetraploid peanuts (Fig. 1D). This finding suggests that changes in DNA methylation are not regulated by the methyltransferases responsible for maintaining methylation levels. However, the demethylases ROS1b exhibited higher expression levels in diploid than tetraploid (Fig. 1D), aligning with the trend of higher methylation levels in CG and CHG in tetraploid compared to diploid. This implies that the elevated methylation levels of CG and CHG are attributed to the reduced expression of demethylases. In contrast, CHH methylation displayed an inconsistent trend compared to CG and CHG due to the unique structure of the CHH background. CHH methylation primarily originates from de novo methylation through the RdDM pathway. To further explore this, we identified key genes involved in the RdDM pathway in peanuts and analyzed their expression. We found that *AGO4* and *DRM2* in the pathway showed the same trend of CHH methylation changes, suggesting that the changes in CHH methylation during peanut evolution were mainly regulated by the de novo methylation through the RdDM pathway (Fig. 1D). Furthermore, we validated the accuracy of our RNA-seq data through RT-qPCR analysis of the key genes mentioned above. The RT-qPCR results were consistent with the RNA-seq data for the selected genes, confirming the high accuracy and reproducibility of our RNA-seq data set (Supplementary Fig. S2, A to E).

### Variation in DNA methylation between diploid and tetraploid peanuts in collinear sequences

**Figure 2.**
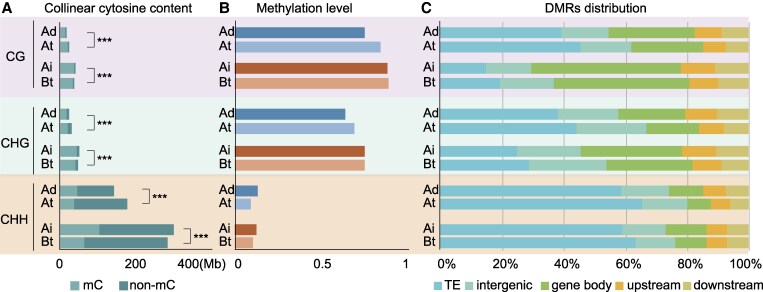
Comparison of DNA methylation in the collinear region of diploid and tetraploid peanuts. **A)** Comparison of cytosine content in the collinear region of all samples. *** indicates *P* < 0.001 for χ² test. **B)** Global DNA methylation level in the collinear region of all samples. **C)** Distribution of DMRs in the collinear region.

During the evolutionary process, the peanut genome undergoes sequence variation. To eliminate the influence of sequence variation on overall genomic methylation, as well as to refine methylation differences between peanuts, we identified regions of collinearity between peanut tetraploids and diploids after calculating the overall methylation landscape of the global genome for subsequent comparative DNA methylation analyses. Through collinear analysis, we identified a total of 1.6 GB of conserved regions in tetraploid peanuts. Interestingly, the number of methylation sites varied significantly between tetraploid and diploid peanuts, even within the conserved regions (Fig. 2A; Supplementary Table S3). The methylation levels of these conserved regions were consistent with the global DNA methylation analysis, especially in CHH methylation (Fig. 2B).

To further investigate the DNA methylation differences, we identified differentially methylated regions (DMRs) within conserved regions between tetraploid and diploid peanuts. A total of 614,702 DMRs were obtained between the diploid Ad genome and the tetraploid At subgenome (183,086 CG, 112,372 CHG, and 319,244 CHH). In contrast, approximately half as many DMRs were identified between the Ai and Bt subgenomes as in the A subgenome (103,859 CG, 100,976 CHG, and 126,499 CHH), reflecting the minor differences in DNA methylation between the Ai and Bt subgenome. We analyzed the distribution of DMRs in the genomes, revealing significant differences in the distribution of the 3 contexts of DMRs, as well as in the comparisons between different peanut genomes. It is noteworthy that a substantial proportion of the DMRs between the diploid Ad genome and the tetraploid At subgenome are situated within the TE region, particularly evident in the distributions of CHG and CG DMRs. The proportion of DMRs both upstream and downstream of the gene was relatively low in all 3 contexts. We also found that the proportions of DMRs on TE were all lower in tetraploids than in diploids, and DMRs from ancestral diploid peanuts were more enriched in genetic regions than tetraploid peanuts in all 3 methylation contexts (Fig. 2C), which seems to imply that the different subgenomes of tetraploid peanuts have undergone different methylation variants compared to the ancestral genome.

### Expression bias of homoeologous genes during the evolution of tetraploid peanut

**Figure 3.**
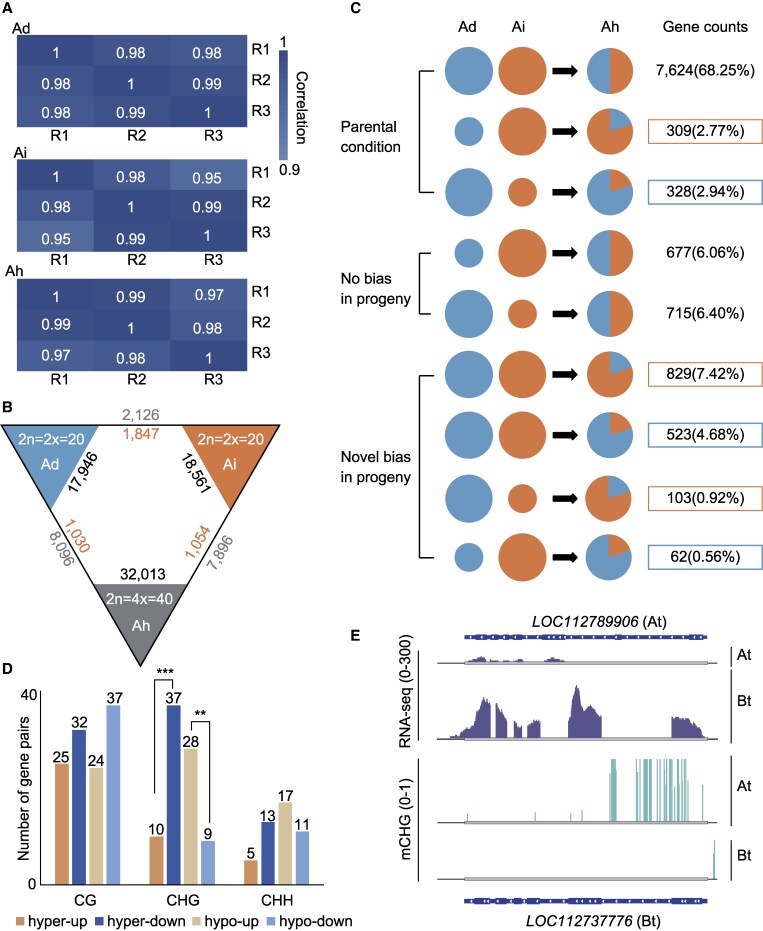
CHG methylation influences gene bias expression in polyploid peanuts. **A)** Correlation between replicate samples of diploid and tetraploid peanuts. **B)** DEGs between tetraploid peanut and its diploid ancestor. The number on the outer of the triangle represents the number of downregulated genes, while the number opposite it represents the number of upregulated genes. **C)** Expression bias of homoeologous genes in diploid and tetraploid peanuts. Circles and fan sizes represent relative expression levels. **D)** The number of homoeologous genes that were differentially methylated in the gene body and showed expression differences. The levels of CG (>0.4), CHG (>0.4), and CHH (>0.1) methylation difference and fold changes of expression (>2-fold) between A and B homoeologs were used as cutoff values. ** and *** represent *P* < 0.01 and 0.001 for the binomial test. **E)** Genome browser showing hypermethylation in the gene body correlating with repression expression in the homoeologous gene pairs.

To investigate the mechanism of DNA methylation in gene transcription during peanut evolution, we isolated RNA samples from leaf tissues of cultivated tetraploid peanut (Ah) and its diploid parents (Ad and Ai). Three biological replicates were sampled for RNA sequencing from each species, resulting in a total of 475,453,244 reads obtained (Supplementary Table S4). Pearson's correlation coefficient between biological replicates within each sample group exceeded 0.95, indicating a robust correlation among these replicates. The above analysis demonstrates that the high quality of our sequencing data (Fig. 3A) can be used for subsequent analysis. We employed FPKM ≥ 1 as the threshold for determining gene expression, revealing 17,946 and 18,561 expressed genes in diploid peanut Ad and Ai, respectively. We detected 32,013 expressed genes in tetraploid Ah, with 15,191 genes from the A subgenome and 16,822 genes from the B subgenome. This observation suggests that certain genes are specifically repressed in tetraploid peanuts relative to their diploid ancestors (Fig. 3B), indicating potential regulatory mechanisms or genetic changes associated with polyploidization.

To explore the genome-wide variation in gene expression during peanut polyploidization, we examined the differential expression of homologous genes between Ah and its ancestral diploids. A total of 18,076 differentially expressed genes (DEGs) were identified, with 9,126 originating from the Ad and At subgenomes and 8,950 from the Ai and Bt subgenomes. Remarkably, during polyploidization, 88.47% (15,992 of 18,076) of the DEGs were downregulated in Ah, and 88.71% (8,096 of 9,126) and 88.22% (7,896 of 8,950) of the downregulated genes were located in the A and B subgenomes, respectively (Fig. 3B). This suggests that the majority of genes in peanut experienced downregulation of expression during polyploidization. We hypothesize that this is due to the increased number of gene copies in tetraploid peanuts and the requirement for homologous genes to maintain important traits in their parents by reducing their expression. Furthermore, genes and their flanking region (2 kb upstream and downstream of the gene body) overlapping with DMRs were designated as differentially methylated genes (DMGs). Interestingly, 81.95% of the DEGs were DMGs compared with the non-DEGs (Supplementary Table S5), and the proportion of DEGs in DMGs (21.77%) was significantly higher than the proportion of DEGs in the whole genome (17.33%) (Supplementary Fig. S3A), suggesting that the variation of DNA methylation may contribute to the differential expression of the homoeologous genes in the process of polyploidization.

Several studies have shown that homoeologous gene pairs may exhibit expression bias in tetraploid during polyploidization, referring to differential expression between homoeologs in different subgenomes (Wu et al. 2018; Li et al. 2020). To investigate the bias of homoeologous gene expression in tetraploid peanuts, we identified a total of 13,750 homoeologous gene pairs in diploid Ad, Ai, and tetraploid Ah, of which 11,170 homoeologous genes were expressed in at least 1 species. We categorized these homoeologous gene pairs based on their expression patterns in progeny relative to parental conditions: Parental Condition (bias consistent with the parental condition), No Bias in Progeny (bias present in the parental but not in the tetraploid), and Novel Bias in Progeny (bias different from parental bias condition). Our analysis revealed 73.96% (8,261 of 11,170 homoeolog pairs) exhibited Parental Condition, which means that more than half of the homoeolog pairs had conserved expression patterns in tetraploid peanut. Whereas 12.46% (1,392) homoeolog pairs showed No Bias in Progeny, suggesting restoration of expression balance compared to parental conditions. Conversely, 13.58% (1,517) of the homozygous gene pairs showed Novel Bias in Progeny, representing an unusual pattern of expression bias distinct from parental patterns (Fig. 3C; Supplementary Fig. S2F). Overall, 80.72% (9,016) of the homoeologous gene pairs exhibited no expression bias in the tetraploid peanut, while the remaining 19.28% (2,154) showed biased expression of gene pairs. This phenomenon is also observed in cotton and oilseed rape, suggesting that most of the homoeologous genes may be conserved during polyploidization. However, the proportion of genes exhibiting balanced expression is higher in peanuts, possibly attributable to species specificity. This disparity might stem from evolutionary factors leading to less intersubgenomic variation in peanuts compared to oilseed rape and cotton, where more subgenomic variation is prevalent (Li et al. 2014; Wu et al. 2018). Notably, among the biased expression homoeologous pairs exhibited in tetraploids, the number of homoeologous gene pairs favoring B subgenome was significantly higher than the number of homoeologous gene pairs favoring A subgenome (A-bias vs. B-bias = 913 [42.39%] vs. 1,241 [57.61%], *P* = 1.68e-12 by binomial test] (Fig. 3C). To explore the functions of the biased-expressed homoeologous gene pairs in tetraploid peanut, we conducted GO enrichment analysis. We found that the homoeologous genes with biased expression in tetraploid peanuts mainly play important roles in root system development, response to stress, and responding to light stimuli and are also involved in fatty acid, nucleotide, organophosphate, and organonitrogen compound metabolic process (Supplementary Fig. S4). In addition, we found that these genes are also enriched by processes such as DNA methylation and mRNA methylation. All these results suggest that homoeolog DEGs play a very important role in the growth of tetraploid peanuts. The key homoeologous genes involved in the important biological process of polyploid peanuts might be dominated by different subgenomes.

### DNA methylation influences homoeologous gene expression bias in polyploid peanuts

To assess the influence of DNA methylation on the expression bias of homoeologous gene pairs in polyploid peanuts, we calculated the methylation levels for the gene body, promoter region (2 kb upstream), and 2 kb downstream of the biased-expressed homoeologous gene pairs. We observed that for gene body, the CG methylation levels of most homoeologous gene pairs were relatively consistent and positively correlated, regardless of the expression levels of the homoeologous gene pairs, while the methylation levels of CHG and CHH exhibited a weak correlation (Supplementary Fig. S5). Among the 2,154 biased-expressed homoeologous gene pairs, we identified 248 pairs that were differentially methylated in CG, CHG, and CHH in the gene body (Fig. 3D). It indicates that there are still certain key genes that may indeed be influenced by DNA methylation in polyploid peanut. We found that only the methylation level of CHG in the gene body was significantly negatively correlated with the differential expression profile, suggesting that CHG methylation in the gene body may have a repressive effect on the expression of homoeologous genes (Fig. 3D; Supplementary Fig. S3B), corroborating our conjecture, and also in agreement with the results of a previous study in cotton (Song et al. 2015). Here, we identified *LOC112789906* on subgenome At and *LOC112737776* on subgenome Bt as a pair of differentially expressed homoeologous genes. These genes encode RNA (sigB) polymerase factors involved in the biosynthesis of organic nitrogen compounds and metabolism processes, protein metabolism, and other important processes in cells. In tetraploid peanut, the gene expression of *LOC112737776* was much higher than that of *LOC112789906*, which was hardly expressed. Interestingly, we observed that the methylation level of CHG in the gene body of *LOC112789906* was substantially higher than that of *LOC112737776*, which exhibited minimal CHG methylation in its gene body (Fig. 3E). Based on these findings, we hypothesized that the hypermethylation of CHG on the gene body may suppress gene expression, leading to expression bias of homoeologous gene pairs in polyploids.

### DNA methylation in peanuts modulates the expression level dominance phenomenon

**Figure 4.**
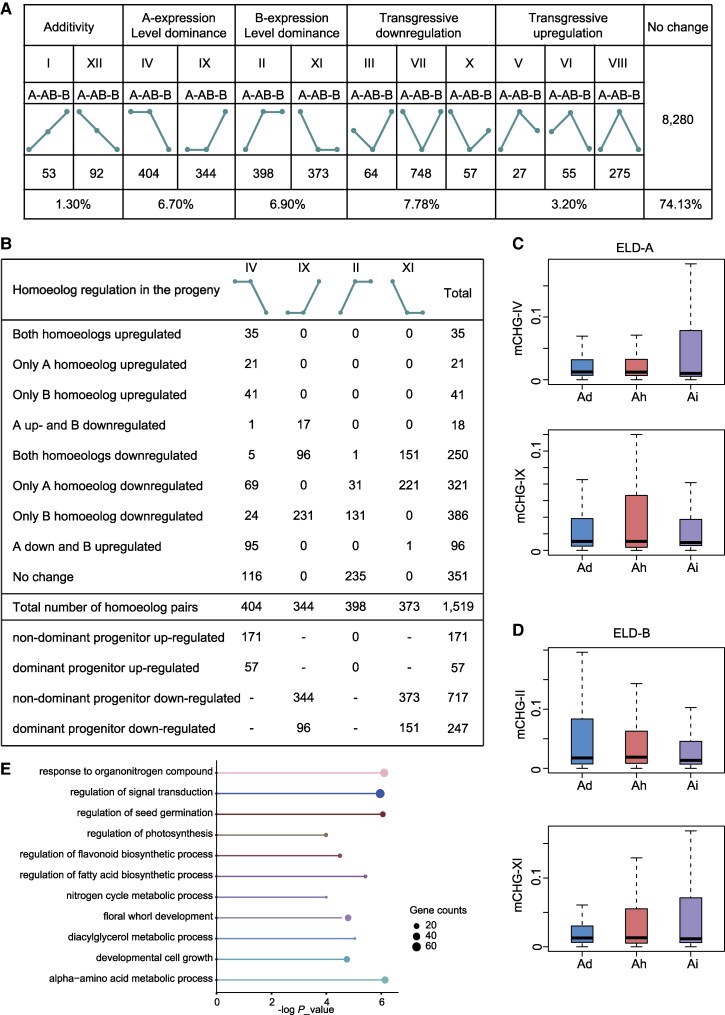
CHG methylation modulates the expression level dominance of subgenomic genes in polyploid peanuts. **A)** Twelve different expression patterns are present in Ah relative to the 2 diploid ancestors. Based on the different expression patterns, genes in Ah and its ancestors were categorized as additively expressed genes, ELD genes, and transgressive expressed genes. A, B, and AB represent Ad, Ai, and Ah peanuts, respectively. **B)** The number of homoeologous gene pairs in ELD. C and D) Box plots of CHG methylation levels of homoeologous gene body in ELD. A-subgenome dominance **C)** and B-subgenome dominance **D)**. The centerline represents the median (50th percentile). The box extends from the 25th percentile to the 75th percentile. The whiskers extend to the most extreme data points within 1.5 times the interquartile range from the edges of the box. **E)** GO enrichment analysis of ELD homoeologous gene pairs in Ah.

In addition to studying the expression bias of homoeologous gene pairs, expression level dominance (ELD) is often employed to investigate heterologous polyploid species. While expression bias mainly analyzes the relative expression levels of homoeologous gene pairs among polyploid subgenomes, ELD compares the total expression of a homoeologous gene pair in a heterozygous polyploid with the relative expression of its parents. Here, we classify tetraploid peanuts into 4 major categories: additivity, transgressive regulation, ELD, and no change, further subdividing them into 12 subcategories depending on the expression pattern to compare the total expression of a homoeologous gene pair in a heterologous tetraploid with that in its diploid parent (Fig. 4A) (Grover et al. 2012; Yoo et al. 2013; Wu et al. 2018; Li et al. 2020).

We found that 74.13% (8,280 in 11,170) of the homoeologous gene pairs showed equivalent expression (no change) in tetraploid peanuts (equivalent expression means that the total expression level of the homoeologous gene pairs in the tetraploid is equal to that in the 2 diploid parents). Additionally, 13.6% (1,519 in 11,170) of the homoeologous gene pairs displayed positional ELD (corresponding to subcategories II, XI, IV, and IX). Among them, the number of A expression-dominant gene pairs (748) was less than that of B expression-dominant gene pairs (771). It was also found that only 145 gene pairs showed additivity expression and 1,226 gene pairs showed transgressive expression, of which 869 were transgressive downregulation and 357 were transgressive upregulation. In summary, most of the homoeologous gene pairs showed either equivalent or dominant expression during polyploidization, probably due to the generation of duplicate copies between genes with similar or redundant functions during the initial merging of A and B subgenomes, leading to altered gene expression patterns (Comai 2005) (Fig. 4A; Supplementary Fig. S2G).

To delve deeper into ELD, we meticulously compared the expression levels of tetraploid peanut with those of its diploid parents. Our analysis revealed that in 76.89% (=(1,519 − 351)/1,519) exhibiting ELD-A (A subgenome is the dominant progenitor) and ELD-B (B subgenome is the dominant progenitor), the main reason for the occurrence of ELD was the expression modification of at least one of the homozygous genes upon merging of the A and B subgenomes, thereby resulting in ELD (Fig. 4B). We found that most of the homoeolog expression modifications were downregulated during polyploidization, with the number of single homoeologous genes downregulated or both homoeologous genes downregulated being 707 (=321 + 386) and 250 pairs, respectively. In addition, we observed fewer expression modifications for gene pairs involving A homoeologs (741 genes = 35 + 21 + 18 + 250 + 321 + 96) compared to those involving B homoeologs (826 genes = 35 + 41 + 18 + 250 + 386 + 96). For gene pairs categorized in subcategories IX and XI, where the expression of the dominant parent was lower than that of the nondominant parent, this phenomenon could mainly arise from the downregulation of homoeologous genes in the nondominant parent (171 in 228 genes, *P* = 2.02e-14 by binomial test) (Fig. 4B). Conversely, for gene pairs in IV and II, where the expression of the dominant parent was higher than that of the nondominant parent, this could be explained by the upregulation of homoeologous genes from nondominant parents (717 in 964 genes, *P* < 2.2e-16 by binomial test) (Fig. 4B). Taken together, this suggests that up-/downregulation of homoeologous genes from nondominant progenitors is the primary cause of dominant gene expression, which is consistent with previous findings in *B. napus* (Li et al. 2020).

To investigate the impact of DNA methylation on ELD genes, we calculated the methylation levels of the gene body and its upstream and downstream 2-kb regions for all ELD genes (Supplementary Fig. S6). We found that the CHG methylation levels of all 4 groups of ELD genes in the gene body region showed a completely consistent pattern of negative correlation with their expression levels (Fig. 4, C and D), suggesting that CHG methylation influences the dominant expression of homoeologous genes during peanut polyploidization. Meanwhile, we performed GO enrichment analysis and found that ELD homoeologous gene pairs in tetraploid peanut were mainly involved in response to organonitrogen compound, regulation of seed germination and photosynthesis, regulation of flavonoid biosynthetic process and fatty acid biosynthetic process, and other important biological functions (Fig. 4E). In summary, we identified the dominantly expressed genes in tetraploid peanut after polyploidization, which inherited the expression pattern of the ancestral diploid. It was also found that these key genes occurred in ELD because the gene body was modified by CHG methylation, which provided an important basis for revealing the homoeologous gene evolution pattern of polyploid species.

### Regulation mechanism of DNA methylation on peanut seed subgenomes

**Figure 5.**
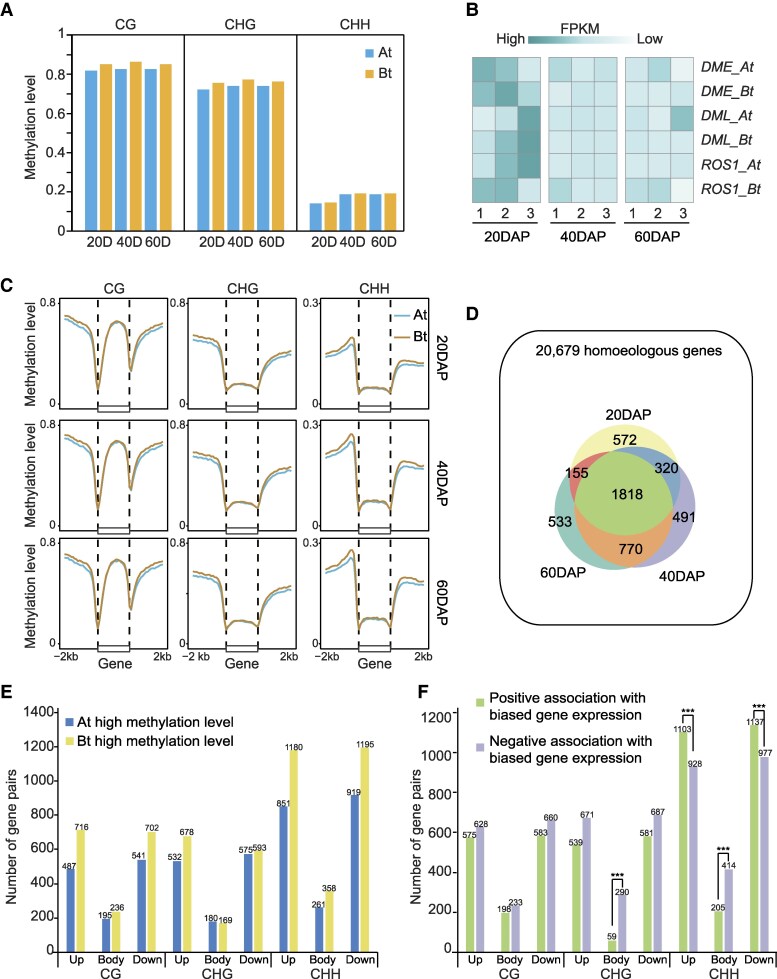
DNA methylation regulates the subgenome of tetraploid peanut seeds. **A)** Global methylation levels for subgenomes of tetraploid peanut at 3 stages of seed development in combined biological samples. 20D, 40D, and 60D represent 20, 40, and 60 d after peanut pegging, respectively. **B)** Heatmap showing expression levels of demethylases. **C)** Metaplot showing gene methylation profiles in tetraploid peanut subgenomes in all contexts. **D)** Venn plot showing the number of biased gene pairs at 3 developmental periods. **E)** Barplot represents the number of biased gene pairs associated with DNA methylation differences. **F)** Barplot showing the numbers of positive/negative association relationships for DNA methylation and biased expression of homoeologous gene pairs. *** represents *P* < 0.001 for the binomial test.

Epigenetic inheritance, particularly DNA methylation, plays a crucial role in plant growth and development, with a significant impact on seed development (Initiative 2000; Gapper et al. 2013; Matilla 2020). Building upon our exploration of DNA methylation's role in peanut polyploidization, we similarly investigated its regulatory role in the development of tetraploid peanut seeds. We performed WGBS of 2 biological replicates and RNA-seq of 3 biological replicates for 3 periods of seed development 20 DAP, 40 DAP, and 60 DAP (DAP: day after pegging) in tetraploid peanut Ah. The sequencing quality was good enough for subsequent analyses (Supplementary Tables S1 and S4 and Fig. S7). We initially compared the overall methylation differences between the 2 subgenomes of tetraploid peanut. Our findings revealed that throughout the 3 developmental stages of peanut seeds, in all 3 contexts, Bt exhibited higher methylation levels compared to At, which aligns with our prior findings. Additionally, across the 3 developmental stages, only CHH methylation demonstrated a significant increase from 20 to 40 DAP, while CG and CHG methylation remained largely unchanged (Fig. 5A). Through an analysis of the methyltransferases and demethylases influencing DNA methylation changes, we observed that the decreased expression of demethylases contributed to the elevated levels of CHH methylation (Fig. 5B). Concurrently, we observed a consistent trend of Bt exhibiting greater methylation levels compared to At in the genes of the subgenome, mirroring the genome-wide methylation patterns (Fig. 5C).

In tetraploid peanut, we identified 4,668 out of 20,679 homoeologous gene pairs exhibiting bias during seed development (Fig. 5D). To investigate how DNA methylation influences the bias phenomenon of homoeologous genes in seeds, we computed methylation levels within the gene body and the upstream and downstream regions (2 kb) of all biased gene pairs. Our analysis revealed that the methylation levels of the majority of biased gene pairs showed higher methylation in Bt compared to At, except for the CHG body region of DMG pairs, which exhibited higher methylation in At than Bt (Fig. 5E). This finding is consistent with our previous observations suggesting that CHG methylation affects the expression bias of subgenomic homologous gene pairs. Further analysis of the direction of DNA methylation differences and expression bias for biased gene pairs unveiled a significant negative correlation between CHG and CHH methylation and expression within the gene body region (Fig. 5F). Moreover, the upstream and downstream regions of CHH also demonstrated a significant positive correlation, echoing results from a previous study on polyploid walnut (Li et al. 2023a).

Through GO enrichment analysis of biased genes significantly regulated by CHG and CHH, we discovered their involvement in crucial biological functions such as seed development, cation homeostasis, abscisic acid transport, triglyceride biosynthetic process, and fatty acid elongation (Supplementary Fig. S8A). For instance, *Diacylglycerol O-acyltransferase 2D-like*, a pivotal gene in triglyceride synthesis, represents a homoeologous pair in peanut. It remained silent in the At subgenome throughout all 3 seed development periods, while exhibiting high CHG methylation in its gene body region. Conversely, it was highly expressed in the Bt subgenome across all 3 periods with minimal CHG methylation observed in its gene body region (Supplementary Fig. S8B), indicating that its expression is governed by CHG methylation within the gene body. In summary, our findings highlight the significance of CHG methylation in the gene body as a crucial factor contributing to the expression bias of polyploid homoeologous gene pairs. Concurrently, CHH may also play a vital role in peanut seed development.

### DNA methylation modifications at different developmental stages of polyploid peanut seeds

**Figure 6.**
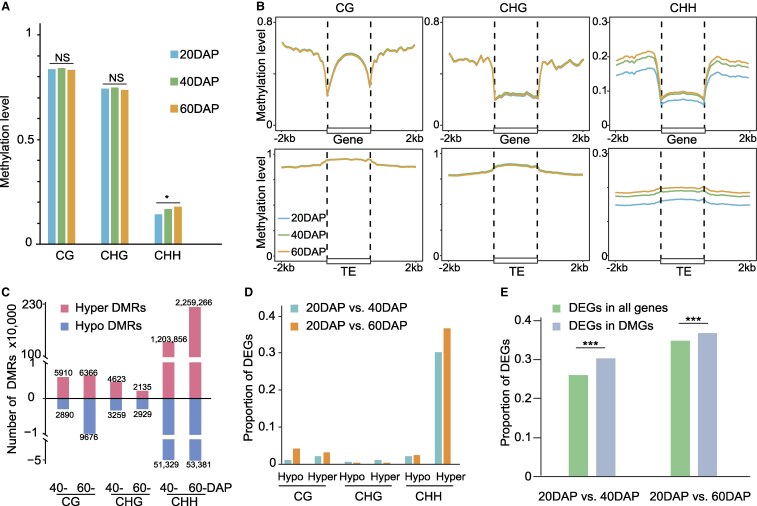
CHH methylation affects seed development in tetraploid peanut. **A)** Global methylation levels of tetraploid peanut in combined biological samples at 20DAP, 40DAP, and 60DAP. * represents *P* < 0.05 for the Student's *t*-test. **B)** Metaplots representing DNA methylation pattern for gene and TE in tetraploid peanuts. **C)** Barplot showing the number of DMRs at 40DAP and 60DAP compared to 20 DAP in all contexts. **D)** The proportion of DEGs associated with DMRs. **E)** The proportion of DEGs in all genes and DMGs. *** represents *P* < 0.001 for the χ² test. DAP, days after peanut pegging; TE, transposable element; DMR, differentially methylated regions; DMG, differentially methylated genes.

Initially, we compared the global methylation levels at different developmental periods and found that the CG and CHG contexts remained essentially constant during seed development. In contrast to the regulatory mechanisms during peanut evolution, CHH methylation may have a significant function in the regulation of peanut seed development. We observed a significant elevation in CHH methylation levels from 20 to 40 DAP, with a slight further increase from 40 to 60 DAP (Fig. 6A). This disparity in regulatory mechanisms between the 2 processes underscores the complexity of biological regulation, suggesting distinct epistatic regulatory models at play during seed development. We further compared the methylation levels of the protein-coding genes and TE in the body and their flanking regions at different developmental periods. Similar to global methylation trends, CG and CHG methylation levels remained unchanged across developmental periods and CHH methylation levels increased from 20 to 40 DAP and 60 DAP (Fig. 6B).

During the development of peanut seeds, there were a total of 3,969 DEGs from 20 to 40 DAP, 6,903 DEGs from 20 to 60 DAP, and only 77 DEGs from 40 to 60 DAP (Supplementary Table S6). This indicates that from 40 to 60 DAP, the genome did not undergo any significant changes, both in terms of gene expression and methylation levels. Therefore, we explored the local changes of DNA methylation during peanut seed development by comparing 40 and 60 DAP with 20 DAP to identify DMRs. As we expected, the number of DMRs in CHH-hyper is much higher than other types (Fig. 6C). Meanwhile, the number of CHH-hyper-associated DEGs was also much higher than other types (Fig. 6D). We also detected a significantly higher proportion of DEGs associated with CHH-hyper DMRs than the proportion of DEGs in the whole genome (Fig. 6E). In conclusion, it indicates that there is an important regulatory role of elevated CHH methylation on gene expression during peanut seed development.

We observed substantial changes in CHH methylation during seed development in polyploid peanuts, with DMRs predominantly occurring in the CHH context. CHH methylation is known to exert a significant influence on gene expression during fruit development in plants (Huang et al. 2019; Ji et al. 2019). In this study, we identified DEGs associated with CHH-DMRs, and GO functional enrichment analysis of these genes revealed that this fraction of DMGs is involved in seed development, fatty alcohol metabolic, nutrient levels, and hormone levels response (Supplementary Fig. S9A). Among these important DMGs, there is an important transcription factor, WRINKLED1 (WRI1). It has been demonstrated that the WRI1 also affects the fatty acid content of mature seeds of maize, as well as increases the content of some amino acids, amino acid biosynthesis-related compounds, and the products of the tricarboxylic acid cycle. The function of WRI1 is not specific to organisms such as *A. thaliana* and maize, but also exists in rice and peanut plants (Wu et al. 2014). Given that oil content is a crucial trait in peanut breeding, WRI1 is also a key gene regulated by CHH methylation. Elevation of CHH methylation levels in the promoter region of WRI1 resulted in reduced levels of its expression (Supplementary Figure S9B). We found other genes regulated by *WRI1* based on protein interactions (Supplementary Fig. S9C), in which genes regulated by *WRI1* are also involved in processes such as seed and fruit development in peanuts. In summary, we found that CHH methylation is mainly altered during peanut seed development and regulates many key genes involved in important biological processes. We anticipate that our study will serve as a valuable epigenetic resource for future peanut breeding and the enhancement of polyploid crop traits.

## Discussion

DNA methylation is a fundamental regulatory mechanism in numerous biological processes, contributing significantly to the development of various diseases in humans and animals. In plants, DNA methylation also plays a crucial role in evolution and environmental adaptation (Weigel and Colot 2012; Yu et al. 2023, 2024). Allotetraploid peanuts, arising from the hybridization of 2 wild diploid peanut species approximately 9,400 yr ago, offer a compelling opportunity to explore the genome-wide dynamics of DNA methylation during polyploidization. In this study, we meticulously investigated the genome-wide DNA methylation variation in different peanuts during polyploidization (Fig. 1A). To account for the potential impact of sequence variations on DNA methylation patterns during polyploidization, we conducted an analysis focusing on conserved sequences (Fig. 2B). Encouragingly, our findings were consistent with the observed global methylation levels, indicating a high degree of conservation in DNA methylation patterns in polyploid species, a phenomenon also observed in other crops such as cotton (Song et al. 2017) and wheat (Yuan et al. 2020). Our analysis revealed an increase in CG and CHG methylation levels, coupled with a decrease in CHH methylation levels in both subgenomes during polyploidization. From the analysis of the genic region and TE region (Fig. 1C), it was identified that TE might be an important factor in maintaining the global methylation level of the genome. For the part of the genic region that is inconsistent with the global methylation level of the genome, we hypothesized that this could be related to common phenomena in polyploidy such as expression bias of homoeologous genes and dominant gene expression.

Genome-wide duplication and polyploidy are critical drivers of plant evolution and contribute significantly to species diversity. Genome merging or doubling in heterologous polyploids is associated with many biological processes, including sequence elimination (Skalická et al. 2005; Yu et al. 2025), epigenetic modifications (Rapp and Wendel 2005), and activation of genes and TEs (Grover et al. 2012). These processes collectively impact the interactions between 2 or more subgenomes, potentially leading to differences in expression patterns of duplicate genes (Rapp et al. 2009; Jackson and Chen 2010). In this study, we used homoeolog expression bias and ELD to characterize the changes in the expression patterns of homoeologous genes in polyploid peanuts. Homoeolog expression bias is the high expression of 1 homoeologous gene relative to another in polyploid peanut. This phenomenon has been studied in many polyploids, especially in wheat (Akhunova et al. 2010; Li et al. 2014; Powell et al. 2017), cotton (Yoo et al. 2013; Zhang et al. 2015), and oilseed rape (Wu et al. 2018; Li et al. 2020). A total of 11,170 expressed homoeologous gene pairs were identified in our study, of which 11.1% showed expression bias (Fig. 3C), and a higher number of genes biased toward subgenome B. This percentage is lower than that of polyploid species such as cotton and oilseed rape but is consistent with the results of Bertioli et al. in peanut (Bertioli et al. 2019). ELD refers to the total expression of homoeologous gene pairs in polyploids compared with their parents, a concept introduced by Grover et al. and also referred to as “genomic dominance” (Rapp et al. 2009; Grover et al. 2012). Our study marks the exploration of ELD in polyploid peanuts, revealing that 24.1% of the 11,170 expressed homoeologous gene pairs exhibited ELD in polyploids (Fig. 4A), with a higher number of dominant pairs in A subgenome. This phenomenon may have maintained the expression balance of the 2 subgenomes. Moreover, our study integrated DNA methylation with typical polyploid genomic features such as expression bias and ELD. We discovered a significant negative correlation between CHG methylation and homoeologous gene expression, suggesting that CHG methylation may serve as an important epigenetic modifier influencing the expression bias of evolutionary subgenomes in polyploid species. This finding provides insights into epigenetic modifications in polyploid species, offering valuable resources for future studies on gene expression and evolutionary dynamics.

The critical role played by DNA methylation in plant development has been extensively examined. In our investigation, we focused on DNA methylation-related dynamics during peanut seed development. Notably, we observed significant alterations only in CHH methylation during the development of polyploid peanuts, which is consistent with recent peanut studies (Li et al. 2023b). Whether this variation exists in wild diploid peanut seeds or other peanut species urgently needs to be investigated. These studies are uniquely valuable for exploring polyploid genome evolution, crop domestication, and genome-assisted improvement of peanut production (Abdul Aziz and Masmoudi 2024). It is known from previous related research that many epistatic alleles cause morphological and physiological changes during crop evolution and domestication (Cubas et al. 1999; Weigel and Colot 2012). Similarly, during peanut seed development, we found that epigenetic alleles are involved in important biological processes, such as some important genes involved in lipid synthesis, vitamin and nitrogen compound metabolism, etc. These findings offer valuable insights into the role of epigenetic modifications in the evolution of polyploid species and provide genetic foundations for breeding programs aimed at enhancing desirable traits in peanuts. Overall, our study furnishes a significant theoretical framework and empirical evidence for understanding the impact of epigenetic mechanisms on polyploid species’ evolution. Furthermore, it offers avenues for research into the evolutionary dynamics and genetic improvement of polyploid crops like peanuts.

## Materials and methods

### Plant tissue collection and sequencing

The peanut materials used in this study were collected in Fujian, China. Leaf tissues were collected for wild diploid peanuts, and leaf and seed (20, 40, and 60 DAP) tissues were collected for cultivated tetraploid peanuts. After the collection of peanut tissues, samples were rapidly frozen in liquid nitrogen and stored at −80 °C. DNA was obtained from peanut tissues using the Qiagen DNeasy Plant Mini Kit. BS-seq libraries were prepared using the Illumina TruSeq Nano DNA LT kit (San Diego, CA, USA). Two libraries were constructed for each tissue type, which represents 2 biological replicates. Sequencing was performed on an Illumina HiSeq X Ten (San Diego, CA, USA) system according to the manufacturer's instructions, yielding a paired-end read length of 150 bp. Total RNA from peanut samples was extracted using cetyltrimethylammonium bromide, and then, mRNA was enriched using magnetic beads with oligo (dT). All cDNAs were reverse transcribed and sequencing libraries were then constructed using the VHTS Universal V6 RNA-seq Library Prep Kit (Illumina, San Diego, CA, USA). Sequencing libraries used the paired-end 150-bp method were sequenced on the Illumina Novaseq 6000 (San Diego, CA, USA) platform.

### Whole-genome bisulfite sequencing and analysis

Whole-genome bisulfite sequencing reads were quality-controlled by Trimmomatic v0.39.1 (Bolger et al. 2014). The tetraploid peanut (Bertioli et al. 2019) and 2 diploid peanuts (Bertioli et al. 2016) reference genomes used in this study were downloaded from the NCBI database (accession number: GCF_000817695.2 for Ad, GCF_000816755.2 for Ai, GCF_003086295.2 for Ah). Genome annotation files were downloaded from PeanutBase (https://www.peanutbase.org). The clean reads were then mapped to the corresponding peanut reference genome using BSMAP v2.902 (Xi and Li 2009), allowing a maximum of 6 base mismatches per read, and finally, only uniquely compared reads were retained for subsequent analysis. The methylation level of each cytosine was next extracted using the script methratio.py. In addition, the conversion rate was calculated from the phage genome. ViewBS v0.1.9 (Huang et al. 2018) was used to calculate the global methylation level of the genome and the methylation level of the genes. The methylation level was calculated using the weighted methylation level (#C/#C+#T) (Schultz et al. 2012).

### DMR analysis of conserved regions in peanuts

For the identification of DMRs among peanut subgenomes, we first performed a genomic collinear comparison by using the Last software (Frith et al. 2010). We extracted the regions with a score > 2,000 as the collinear regions. Methylcytosine in the collinear region was identified by binomial distribution test. We cut the collinear regions into 100 bp windows to calculate the DMRs by ANOVA. In defining differential methylation, the difference of CG and CHG was greater than 0.4, and the difference of CHH was greater than 0.1.

### Transcriptome sequencing and analysis

Transcriptome sequencing data were quality controlled using Trimmomatic v0.39, and then, clean data were mapped to the corresponding peanut reference genomes using the software HISAT2 v2.2.1 (Kim et al. 2019). Only uniquely mapped data were retained for subsequent analysis. FPKM was calculated using Stringtie v2.1.7 (Kovaka et al. 2019) to estimate gene expression level. DEGs were characterized using DESeq2 v1.32.0 (Love et al. 2014). The fold change and FDR values of DEGs were taken as 2 and less than 0.05, respectively. We used the trimmed mean of M values (TMM) method in the egdeR package to normalize the expression levels among the different species before calculating them (Robinson and Oshlack 2010).

### Analyses of homoeolog expression bias and ELD

To investigate homoeologous gene expression bias and ELD, we identified homoeologous gene pairs in tetraploid peanut and ancestral diploid by BLAST and MCScanX (Wang et al. 2012). In the expression bias analysis, the expression levels of each homoeologous gene pair were compared and also analyzed by statistical tests using *t*-test. In ELD analysis, we compared the sum of the expression levels of homoeologous gene pairs in tetraploids with the expression levels of ancestral homoeologous genes and also analyzed them by statistical test using *t*-test. For the categorization of biased and dominantly expressed genes, we borrowed from Rapp et al.’s (2009) and Wu et al.’s (2018) research.

### Gene Ontology enrichment analysis

We functionally annotated the protein sequence files of the genes using the eggNOG-mapper (Huerta-Cepas et al. 2019) online tool and then analyzed the enrichment using the GOATOOLS (Klopfenstein et al. 2018) software.

### DEG validation by RT-qPCR

The relative expression level of DEGs was performed using TB Green *Premix Ex Taq* II (ROX Reference Dye II Premixed) (Takara, Dalian, China), and *Ahactin* was used as an internal reference gene. The primer regions of the actin gene are identical among the 3 species. All reactions were performed on an ABI7500 system with 3 technical replicates (Applied Biosystems, CA). The reaction setup and the PCR program were adhered to the previous study (Zhang et al. 2017). The relative expression level of 3 biological replicates was normalized using the 2−ΔΔCT method (Schmittgen and Livak 2008). All the primers used for RT-qPCR are listed in Supplementary Table S7. The graphs were generated using GraphPad Prism v8.0.0 software (https://www.graphpad.com/).

### Accession numbers

Sequence data from this article can be found in the NCBI database with the accession number PRJNA791974 and CNCB database with the accession number CRA020471.

## Supplementary Material

kiaf254_Supplementary_Data

## Data Availability

The data underlying this article are available in the NCBI Sequence Read Archive database with the accession number PRJNA791974 and CNCB database with the accession number CRA020471.

## Supplementary data

The following materials are available in the online version of this article.

**Supplementary Table S1.** Summary of the BS-seq library sequencing.

**Supplementary Table S2.** Mapping ratio of the WGBS among different peanut varieties.

**Supplementary Table S3.** The number of methylcytosines in the collinear regions of peanut.

**Supplementary Table S4.** Summary of the RNA-seq read mapping.

**Supplementary Table S5.** The number of DEGs and DEGS associated with DMR.

**Supplementary Table S6.** Analysis of genes differentially expressed during seed development.

**Supplementary Table S7.** The primers for RT-qPCR.

**Supplementary Figure S1.** Correlation analysis between the replicates of diploid and tetraploid peanuts.

**Supplementary Figure S2.** Expression profiles of genes detected by RNA-seq and RT-qPCR.

**Supplementary Figure S3.** Statistics on the number of DMR-related DEGs and all DEGs.

**Supplementary Figure S4.** Barplot showing GO enrichment analysis of biased genes in tetraploid peanut.

**Supplementary Figure S5.** Analysis of methylation levels and expression levels in different regions of DEGs.

**Supplementary Figure S6.** Metaplots representing the changes in DNA methylation levels in the ELD gene body regions and the flanking regions of Ad, Ai, and Ah.

**Supplementary Figure S7.** Correlation analysis between the replicates of different developmental stages in tetraploid peanuts.

**Supplementary Figure S8.** Functional analysis of biased genes associated with CHG methylation in tetraploids peanut.

**Supplementary Figure S9.** Functional analysis of CHH-hyper DMR-associated DEGs.

## Dive Curated Terms

The following phenotypic, genotypic, and functional terms are of significance to the work described in this paper:

AGO4 Gramene: AT2G27040
AGO4 Araport: AT2G27040

MET1 Gramene: AT5G49160
MET1 Araport: AT5G49160

TMM Gramene: Method in EdgeR to normalize read counts (trimmed mean of M values)
TMM Araport: Method in EdgeR to normalize read counts (trimmed mean of M values)

NCBI Gramene: https://www.ncbi.nlm.nih.gov
NCBI Araport: https://www.ncbi.nlm.nih.gov

CNCB Gramene: https://www.cncb.ac.cn
CNCB Araport: https://www.cncb.ac.cn
