# Supplementary material for: Epigenetic insights into the domestication of tetraploid peanut
Source: Plant Physiol. 2025 Jun 13;198(3):kiaf254. doi: 10.1093/plphys/kiaf254 (PMC12268262; doi:10.1093/plphys/kiaf254)
Supplement: kiaf254_Supplementary_Data [file kiaf254_supplementary_data.zip › Supplementary Figures S1-9.pdf]

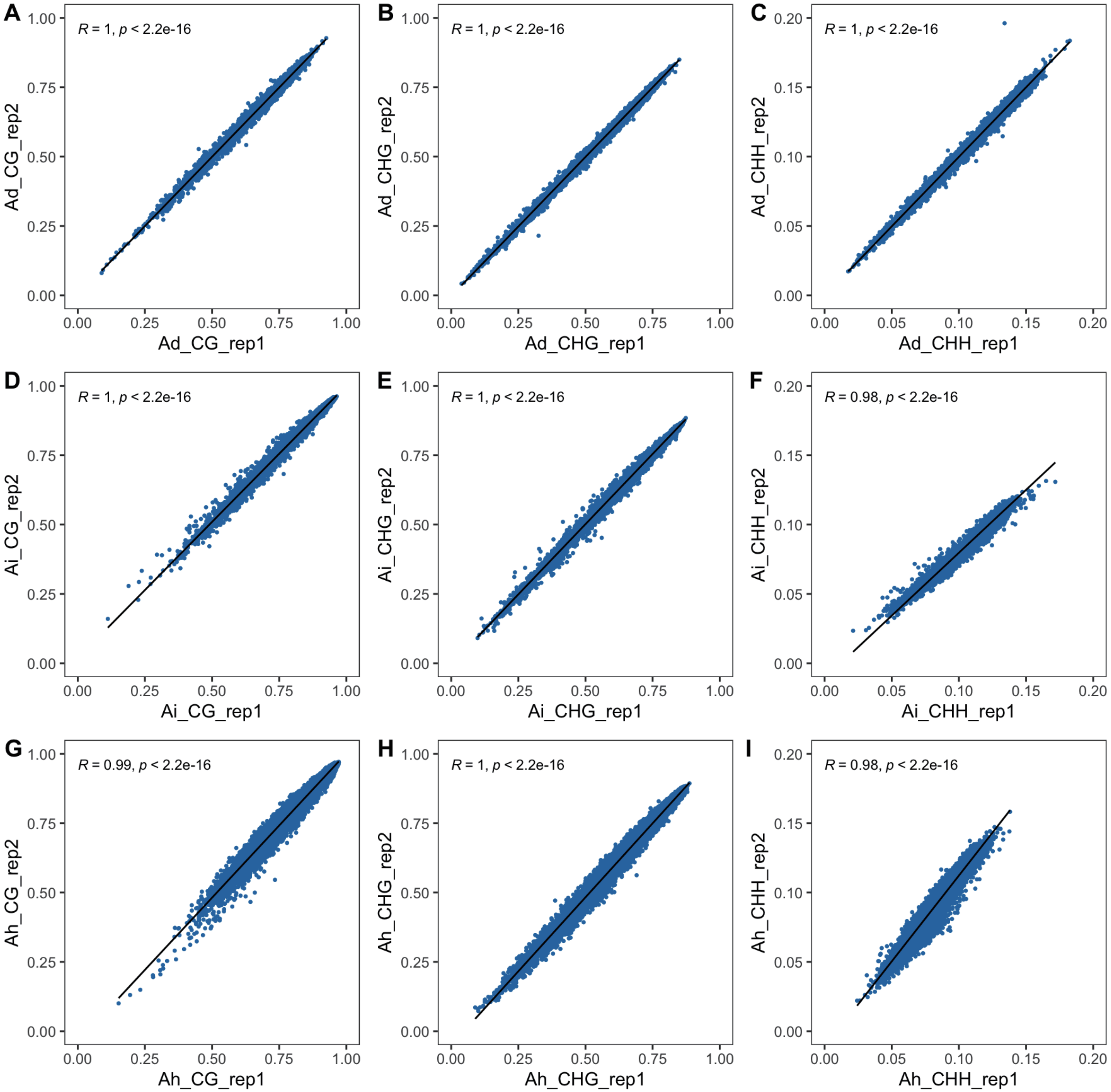

Supplementary Fig. S1. Correlation analysis between the replicates of diploid and tetraploid peanuts. (**A-C**) Correlation between replicates of CG, CHG, and CHH in diploid peanut Ad. (**D-F**) Correlation between replicates of CG, CHG, and CHH in diploid peanut Ai. (**G-I**) Correlation between replicates of CG, CHG, and CHH in tetraploid peanut Ah.

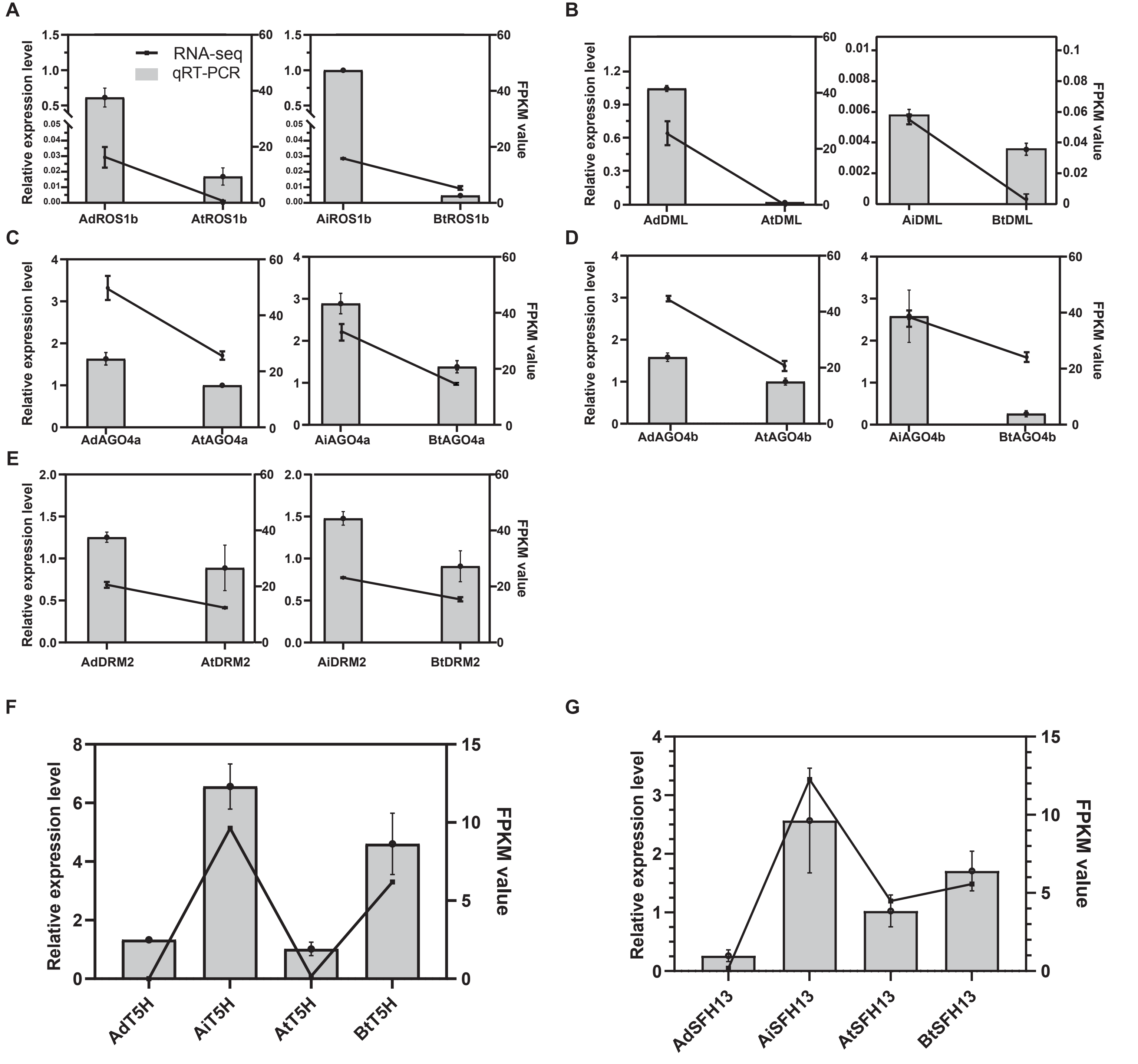

Supplementary Fig. S2. Expression profiles of genes detected by RNA-seq and RT-qPCR. (**A-B**) DNA demethylase. (**C-E**) Genes in RdDM pathyway. (**F**) Bias gene pair. (**G**) ELD gene pair.Three biological replicates were performed for each of these genes, and error bars were labeled using SD.

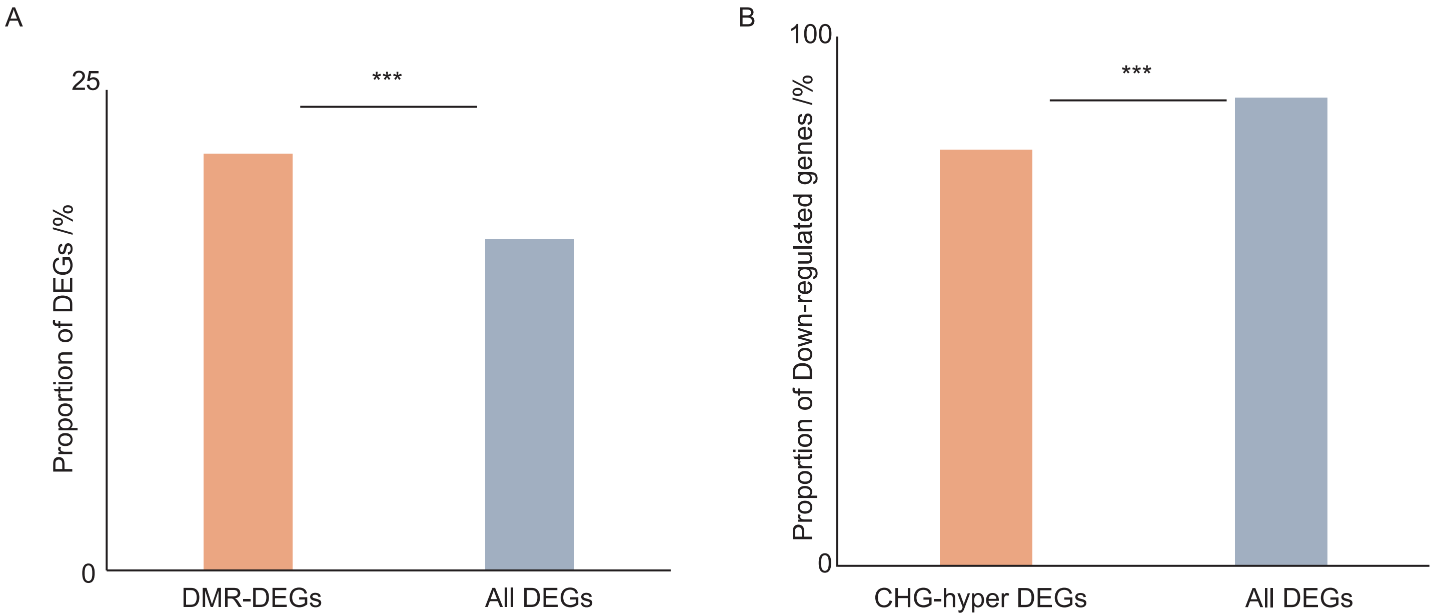

Supplementary Fig. S3. Statistics on the number of DMR-related DEGs and all DEGs. (A) DMR-associated genes are more likely to show differential expression in polyploidization. **(B)** Less of CHG hyper-DMR-associate DEGs show downregulation. ***, P < 0.001 (Chi-square test). Samples were selected from three replicates and error bars were made using SD.

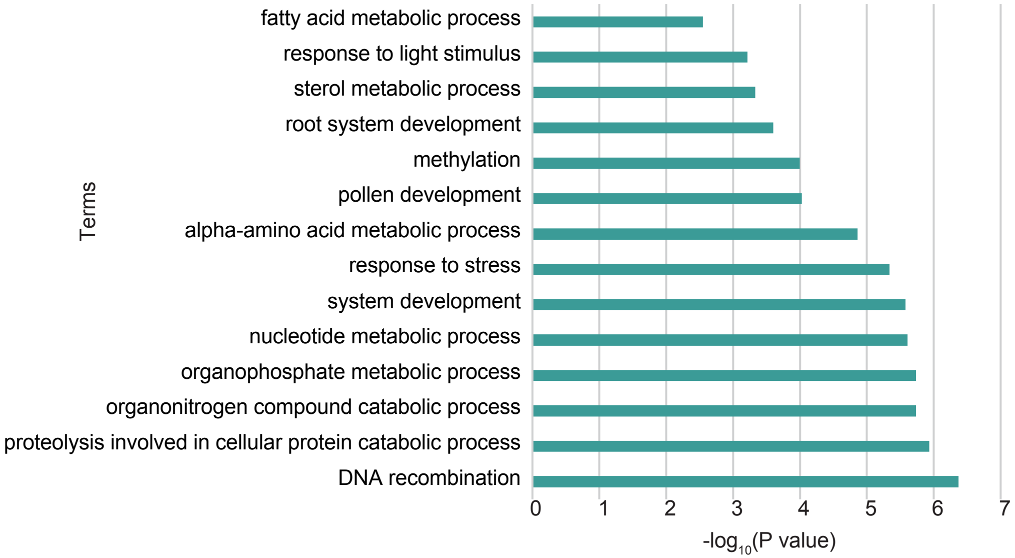

Supplementary Fig. S4. Barplot showing GO enrichment analysis of biased genes in tetraploid peanut.

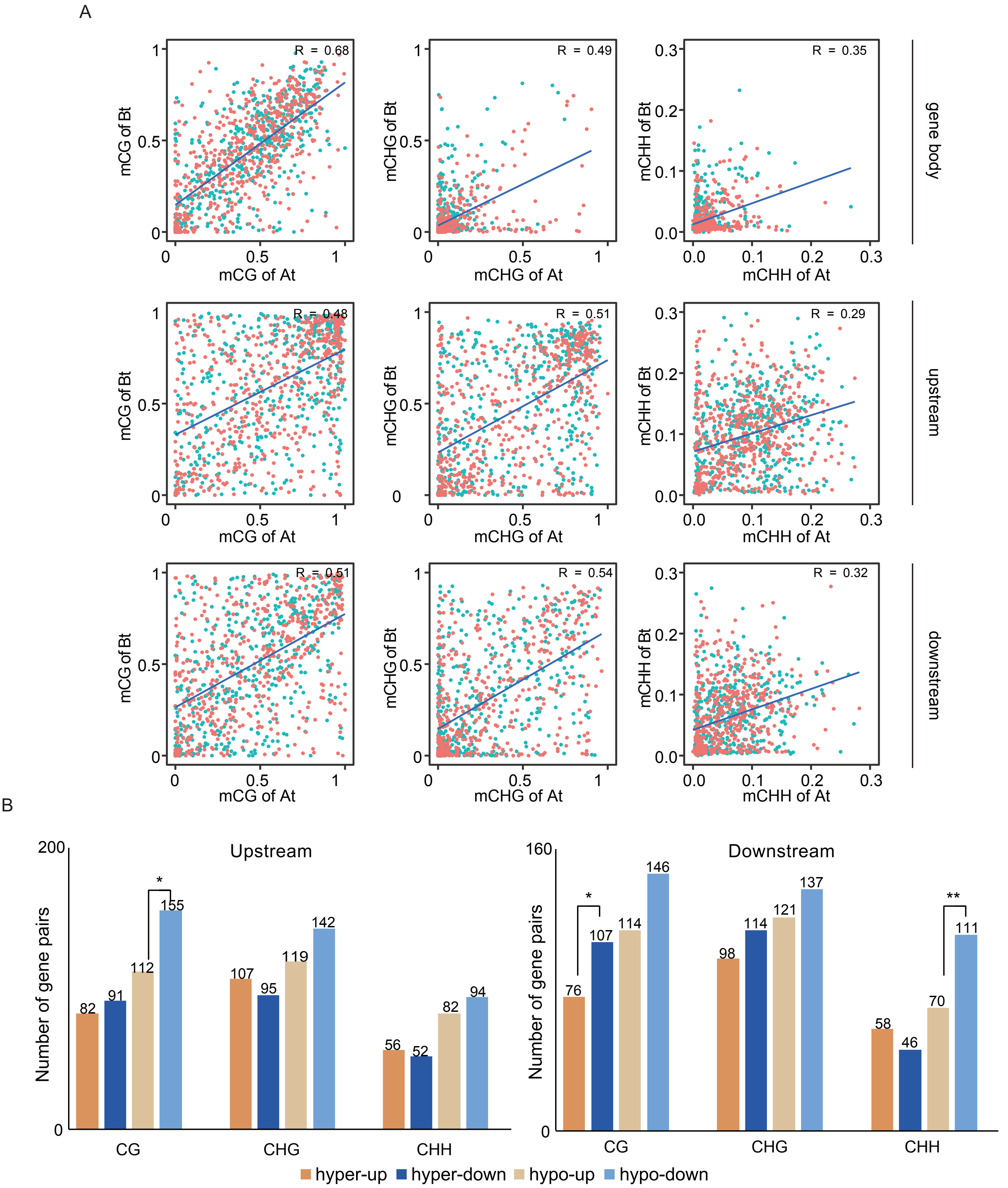

Supplementary Fig. S5. Analysis of methylation levels and expression levels in different regions of DEGs. (A) Correlation map of methylation levels of DEGs among tetraploid peanut homoeologs genes. The cyan dots represent a higher expression level of At than Bt. The red dots represent a less expression level of At than Bt. **(B)** Bar plot of differential methylation of homoeolog DEGs in gene upstream (2 KB) and gene downstream (2 KB). Single and double asterisks indicate a significance level of $P < 0.05$ and 0.01, respectively (Chi-square test).

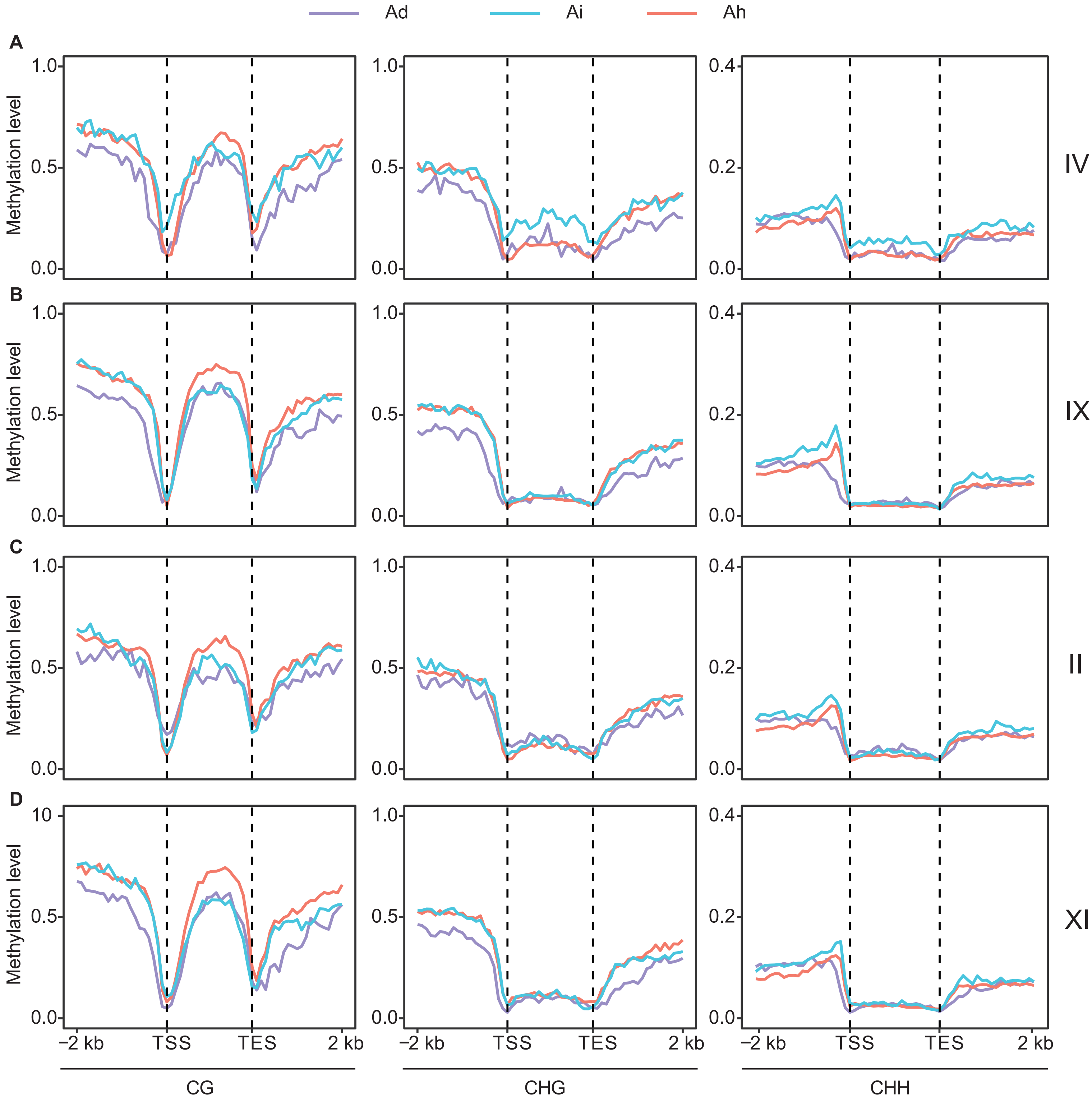

Supplementary Fig. S6. Metaplots representing the changes in DNA methylation levels in the ELD gene body regions and the flanking regions of Ad, Ai, and Ah. (A) Methylation profile of genes in group IV. **(B)** Methylation profile of genes in group IX. **(C)** Methylation profile of genes in group II. **(D)** Methylation profile of genes in group XI.

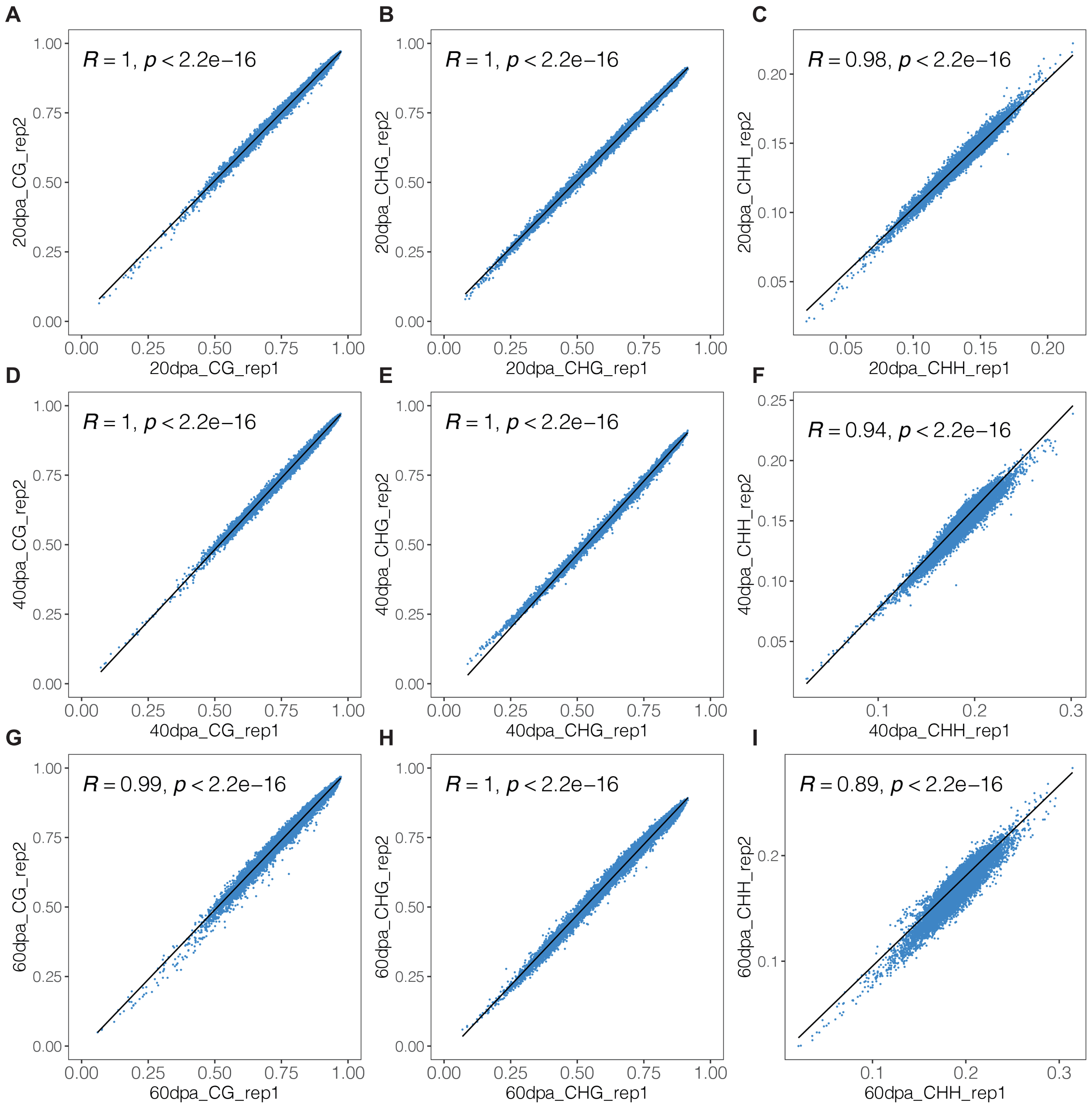

Supplementary Fig. S7. Correlation analysis between the replicates of different developmental stages in tetraploid peanuts. (**A-C**) Correlation between replicates of CG, CHG, and CHH in 20DPA. (**D-F**) Correlation between replicates of , CHG, and CHH in 40DPA. (**G-I**) Correlation between replicates of CG, CHG, and CHH in 60DPA. DPA: Days after peanut pegging.

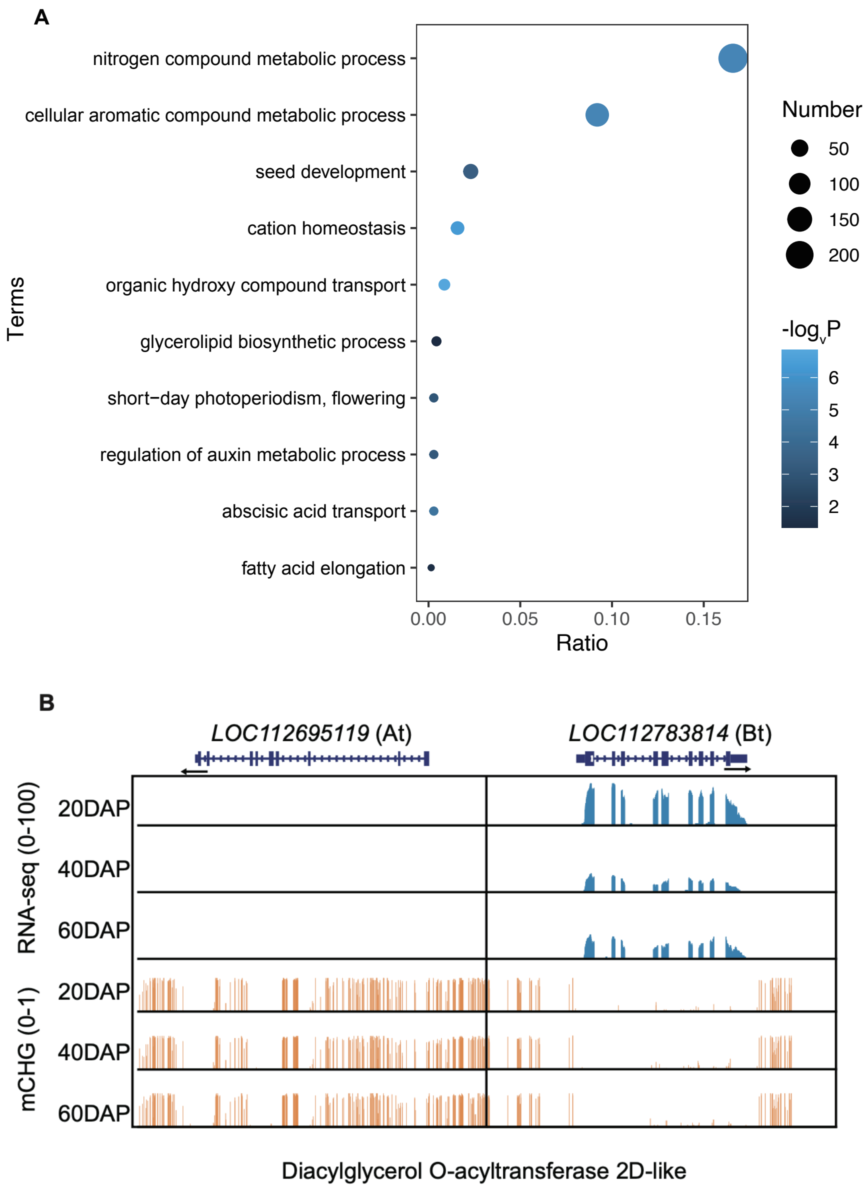

Supplementary Fig. S8. Functional analysis of biased genes associated with CHG methylation in tetraploids peanut. (A) GO enrichment of bias genes associated with CHG methylation in tetraploid peanut. **(B)** Genome browser showing the expression level and CHG methylation level of a pair of homoeologous genes in tetraploid peanut.

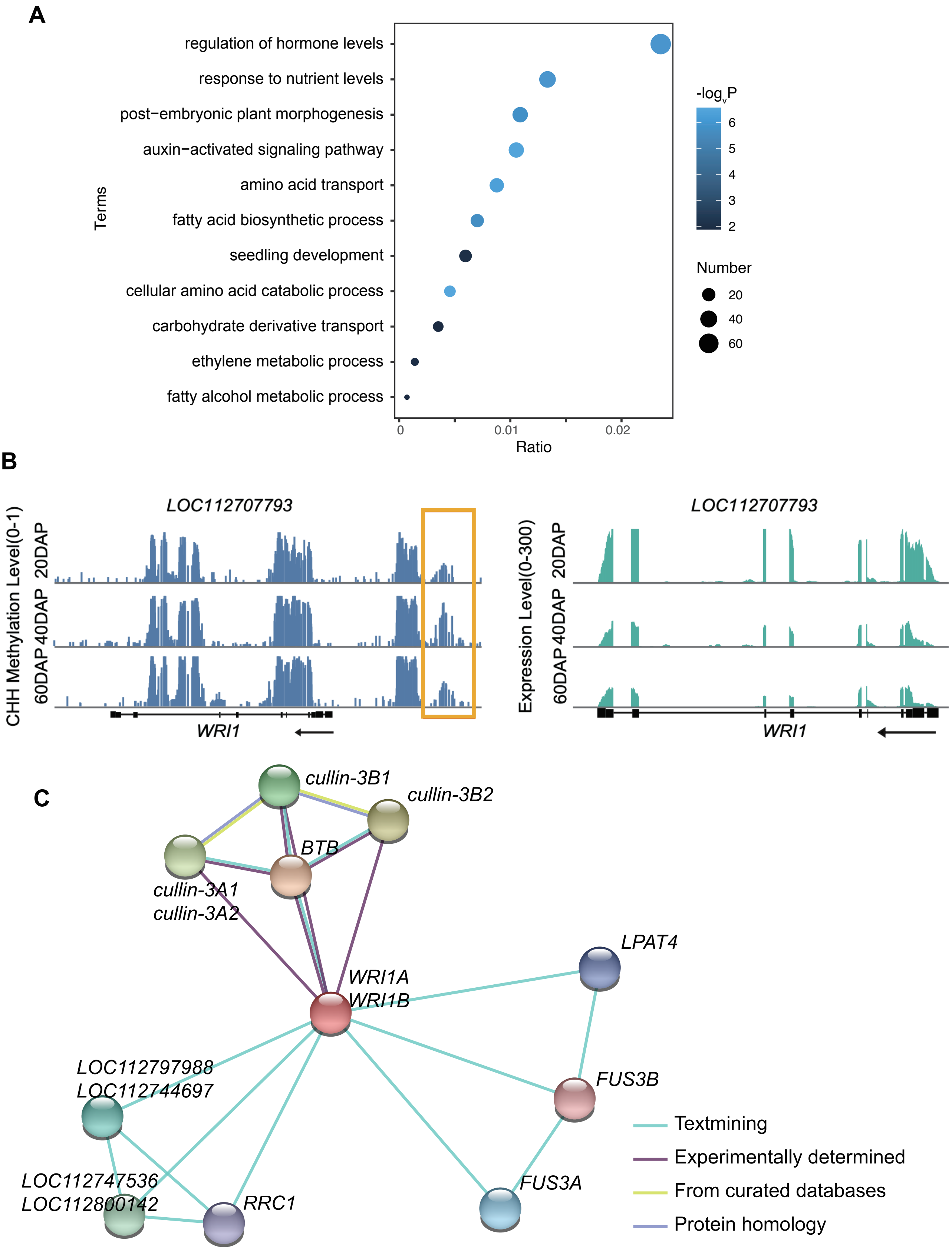

Supplementary Fig. S9. Functional analysis of CHH-hyper DMR associated DEGs. (A) GO enrichment of DEGs associatied with CHH-hyper DMR in tetraploid peanut. **(B)** Genome browser showing the expression level and CHH methylation level for *LOC112707793* (*WRI1*). **(C)** Gene network diagram of interaction with *WRI1*.
