# Supplementary material for: Epigenetic insights into the domestication of tetraploid peanut
Source: Plant Physiol. 2025 Jun 13;198(3):kiaf254. doi: 10.1093/plphys/kiaf254 (PMC12268262; doi:10.1093/plphys/kiaf254)
Supplement: kiaf254_Supplementary_Data [file kiaf254_supplementary_data.zip › Supplementary Tables 1-7.docx]

**Supplementary Table S1. Summary of the BS-seq library sequencing.**

| Tissues | Samples | Total pairs | Unique  pairs | Mapping  ratios | Conversion  rate* | Coverage |
| --- | --- | --- | --- | --- | --- | --- |
| Leaf | Ad01 | 97,664,890 | 43,080,887 | 44.10% | 99.43% | 12.41 |
|  | Ad02 | 101,031,219 | 44,588,298 | 44.10% | 99.48% | 12.84 |
|  | Ai01 | 102,317,277 | 76,951,771 | 75.20% | 99.45% | 17.19 |
|  | Ai02 | 96,173,005 | 71,288,261 | 74.10% | 99.38% | 15.92 |
|  | Ah01 | 99,710,290 | 74,274,441 | 74.50% | 99.31% | 8.78 |
|  | Ah02 | 102,739,864 | 80,415,002 | 78.30% | 99.39% | 9.51 |
| Seed | 20DAP01 | 258,892,908 | 205,133,128 | 79.20% | 99.43% | 24.24 |
|  | 20DAP02 | 304,117,173 | 238,904,143 | 78.60% | 99.42% | 28.25 |
|  | 40DAP01 | 355,905,901 | 281,557,310 | 79.10% | 99.53% | 33.27 |
|  | 40DAP02 | 321,833,956 | 252,765,443 | 78.50% | 99.60% | 29.86 |
|  | 60DAP01 | 257,674,265 | 206,139,905 | 80.00% | 99.54% | 24.36 |
|  | 60DAP02 | 285,063,733 | 222,243,236 | 78.00% | 99.48% | 26.28 |

*Conversion rate: The rate of unmethylated C in the genome that is converted to U when the genome is treated with Bisulfite.

**Supplementary Table S2. Mapping ratio of the WGBS among different peanut varieties.**

| Mapping ratio | Ad | Ai | Ah |
| --- | --- | --- | --- |
| Ad01 | 44.1% | - | 29.9% |
| Ad02 | 44.1% | - | 30% |
| Ai01 | - | 75.2% | 66% |
| Ai02 | - | 74.1% | 64.4% |
| Ah01 | 34% | 48.1% | 74.5% |
| Ah02 | 38.3% | 53.1% | 78.3% |

**Supplementary Table S3. The number of methylcytosines in the collinear regions of peanut.**

| Context | Variety | Methylcytosines | All cytosine | Chi-square test |
| --- | --- | --- | --- | --- |
| CG | Ad | 14,290,908 | 17,613,956 | *** |
|  | At | 19,959,146 | 22,562,857 |  |
|  | Ai | 36,678,451 | 39,631,725 | *** |
|  | Bt | 34,488,621 | 37,209,211 |  |
| CHG | Ad | 17,544,197 | 24,025,417 | *** |
|  | At | 22,831,748 | 29,546,275 |  |
|  | Ai | 44,539,045 | 51,697,135 | *** |
|  | Bt | 40,662,041 | 48,716,373 |  |
| CHH | Ad | 48,558,482 | 143,492,721 | *** |
|  | At | 38,166,426 | 174,904,922 |  |
|  | Ai | 104,107,765 | 298,218,270 | *** |
|  | Bt | 65,138,140 | 280,260,589 |  |

**Supplementary Table S4. Summary of the RNA-seq read mapping.**

| Samples | Total pairs | Uniquely mapped pairs | Mapped ratio |
| --- | --- | --- | --- |
| leaf_Ad01 | 19,800,032 | 13,939,007 | 74.27% |
| leaf_Ad02 | 19,795,242 | 14,338,571 | 76.45% |
| leaf_Ad03 | 19,840,031 | 13,527,284 | 72.07% |
| leaf_Ai01 | 20,025,693 | 17,180,039 | 93.75% |
| leaf_Ai02 | 19,700,113 | 16,884,479 | 93.67% |
| leaf_Ai03 | 19,829,555 | 16,989,232 | 93.64% |
| leaf_Ah01 | 39,680,063 | 32,337,946 | 97.36% |
| leaf_Ah02 | 39,472,379 | 31,789,275 | 96.03% |
| leaf_Ah03 | 39,583,514 | 32,093,719 | 96.56% |
| seed_Ah_20DAP01 | 23,306,194 | 17,571,027 | 75.39% |
| seed_Ah_20DAP02 | 22,005,145 | 16,679,260 | 75.80% |
| seed_Ah_20DAP03 | 22,195,381 | 17,098,348 | 77.04% |
| seed_Ah_40DAP01 | 23,458,837 | 16,960,257 | 72.30% |
| seed_Ah_40DAP02 | 22,995,597 | 17,166,442 | 74.65% |
| seed_Ah_40DAP03 | 23,142,523 | 16,794,552 | 72.57% |
| seed_Ah_60DAP01 | 22,382,883 | 16,602,154 | 74.17% |
| seed_Ah_60DAP02 | 22,949,375 | 17,459,384 | 76.08% |
| seed_Ah_60DAP03 | 21,461,117 | 15,714,259 | 73.22% |

**Supplementary Table S5. The number of DEGs and DEGS associated with DMR.**

| Sample | DEG | CG-DMGs | | CHG-DMGs | | CHH-DMGs | | All-DMGs | |  |
| --- | --- | --- | --- | --- | --- | --- | --- | --- | --- | --- |
|  |  |  |  |  |  |  |  |  |  |  |
| Ad | 1,030  (Up) | 770 | 74.76% | 516 | 50.10% | 764 | 74.17% | 959 | 93.11% |  |
| At |  | 803 | 77.96% | 511 | 49.61% | 712 | 69.13% | 928 | 90.10% |  |
| Ad | 8,096  (Down) | 6,680 | 82.51% | 2,728 | 33.70% | 5,162 | 63.76% | 7,585 | 93.69% |  |
| At |  | 6,359 | 78.54% | 2,500 | 30.88% | 4,710 | 58.18% | 7,264 | 89.72% |  |
| Ai | 1,054  (Up) | 664 | 63.00% | 485 | 46.02% | 589 | 55.88% | 807 | 76.57% |  |
| Bt |  | 778 | 73.81% | 499 | 47.34% | 577 | 54.74% | 873 | 82.83% |  |
| Ai | 7,896  (Down) | 5,706 | 72.26% | 1,328 | 16.82% | 2,281 | 28.89% | 6,379 | 80.79% |  |
| Bt |  | 5,143 | 65.13% | 1,096 | 13.88% | 1,985 | 25.14% | 5,749 | 72.81% |  |
| Total | 18,076 |  |  |  |  |  |  | 14,814 | 81.95% |  |

**Supplementary Table S6. Analysis of genes differentially expressed during seed development.**

|  | 20DAP vs. 40DAP | 20DAP vs. 60DAP | 40DAP vs. 60DAP |
| --- | --- | --- | --- |
| Upregulation | 1801 | 2834 | 22 |
| Downregulation | 2168 | 4069 | 55 |
| All | 3969 | 6903 | 77 |

**Supplementary Table S7.** **The primers for RT-qPCR.**

| Primer Name | Primer Sequence (5'-3') |
| --- | --- |
| AdAGO4a-F | GAGAAGACTTCAATCCTAGGAAC |
| AdAGO4a-R | TCTCCACCCTGACAAGCGGAGGA |
| AiAGO4a-F | GAGAAGACTTCAATCCTAGGAAT |
| AiAGO4a-R | TCTCCACCCTGACAAGCGGAGGG |
| AdAGO4b-F | TGGAGAAGATGTTTGAAAGCATG |
| AdAGO4b-R | GTCTGGCTTTCTCTTCGCAGAAG |
| AiAGO4b-F | TGGAGAAGATGTTTGAAAGCATC |
| AdDML-F | TGCCAATGGATATGGCACCATC |
| AdDML-R | ACCACTGAGTCCACAACTGATC |
| AiDML-F | CAACCTCAACCGAAGAACATGG |
| AiDML-R | ATCACATTGAGAAGGTGCATTG |
| AdDRM2-F | CAGGAAGGCTCTAGAGAACTAT |
| AdDRM2-R | GAATGAATTGCCAAGTGACTTG |
| AiDRM2-F | CAGGAAGGCTCTAGAGAACTAC |
| AiDRM2-R | GAATGAATTGCCAAGTGACTTA |
| AdROS1b-F | GAAGATGAAGACAAGTCCAAAT |
| AdROS1b-R | CCAACATTCACTTGTGATTTGTTA |
| AiROS1b-F | GAAGATGAAGCCACGTCCAAAC |
| AiROS1b-R | CCAACGTTTACTTGTGATTTGTTG |
| AdSFH13-F | TCCAGCATGTTCTGTTGCTTCC |
| AdSFH13-R | GTAGTCCAGTGATTTGTCTCAC |
| AiSFH13-F | TCCAGCATGTTCTGTTGCCGCA |
| AiSFH13-R | GTAGTTCAGTGATTTGTCTCAT |
| AdT5H-F | ATCCTTGAAGCTGTATGTGGG |
| AdT5H-R | GTCTTGGTGAGTAGAGGCTTC |
| AiT5H-F | ATCCTTGAAGCTGTATGTGCA |
| AiT5H-R | ATCTTGGTGAGTAGAGGCTTG |
| Actin-F | GAGGAGAATCAGAAGCAAGTC |
| Actin-R | CATATACAGCATAGCGGCACTC |
